# Metformin ameliorates peripheral neuropathy in diabetic rats by downregulating autophagy via the AMPK pathway

**DOI:** 10.20945/2359-4292-2024-0137

**Published:** 2024-11-06

**Authors:** Fangqin You, Diya Xie, Cheng Li, Lihang Yang, Fengmin Liu

**Affiliations:** 1 Fuzhou First General Hospital Affiliated with Fujian Medical University Department of General Surgery Fuzhou Fujian China Department of General Surgery, Fuzhou First General Hospital Affiliated with Fujian Medical University, Fuzhou, Fujian, China; 2 Fuzhou First General Hospital Affiliated with Fujian Medical University Department of Endocrinology Fuzhou Fujian China Department of Endocrinology, Fuzhou First General Hospital Affiliated with Fujian Medical University, Fuzhou, Fujian, China

**Keywords:** Diabetes, diabetic neuropathy, metformin, autophagy, AMPK

## Abstract

**Objective:**

Diabetic neuropathy (DN) is an important complication of diabetes mellitus. Autophagy is considered to be potentially involved in the regulation of DN. Metformin is broadly utilized in the first-line treatment of diabetes. The present work aimed to assess whether and how metformin exerts protective effects in DN.

**Materials and methods:**

A DN rat model induced by streptozotocin (STZ) was established. Metformin was administered to examine its effect on sciatic nerve pathology, and the possible mechanisms involved in this process were explored.

**Results:**

Morphological damage was observed in sciatic nerve samples from diabetic animals, accompanied by decreased p-AMPK expression and increased LC-3 levels. Notably, metformin ameliorated the morphological changes in the sciatic nerve by downregulating autophagy via p-AMPK upregulation.

**Conclusions:**

These results indicate that metformin attenuates peripheral neuropathy in diabetic rats by regulating autophagy.

## INTRODUCTION

Diabetes mellitus (DM) constitutes currently an important and growing health concern worldwide; this life-threatening disease causes disabilities, requires costly management, and reduces life expectancy (1). An estimated 537 million individuals had DM in 2021, and this number is predicted to rise to 643 million and 783 million by 2030 and 2045, respectively (2). Estimates also show that about 6.7 million individuals aged 20-79 years died from DM-associated causes in 2021 (2). Direct health costs for DM care approximated USD 1 trillion in 2021 and are expected to rise year by year (2).

Diabetic neuropathy (DN) is an important, well-known complication of DM. About half of all individuals with DM, including those with prediabetes and types 1 and 2 DM, develop DN (3). The DN symptoms differ depending on the disease stage. Early DN cases experience mostly pain and hyperalgesia. With disease progression, numbness, muscle weakness, loss of balance, and foot ulcers gradually appear (4). Currently, DN treatments encompass intense glycemic control and pain relief drugs (5). However, a recently published meta-analysis of DN trials suggested that glycemic control confers no benefit to most DN cases (6). In addition, a fast glucose level reduction may induce neuropathic pain, also referred to as treatment-induced neuropathy (6). Therefore, current DN therapies remain insufficient.

Different molecular pathways are involved in DN development. The pathological process of DN is multifactorial, with unknown underpinning mechanisms. Potential mechanisms include the polyol pathway (7), hexosamine pathway (8), protein kinase C (PKC) pathway (9), synthesis of advanced glycation end-products (AGEs) (10), and elevation of proinflammatory cytokines (11). Autophagy may also be involved in DN regulation (12), although the specific relationship between autophagy and DN remains unelucidated.

Metformin is broadly applied in the first-line treatment of type 2 DM. Accumulating evidence reveals that metformin exerts anti-inflammatory effects and improves endothelial function in obesity or DM associated with a high-fat diet (HFD) (13). Meanwhile, previous research has shown that metformin contributes to the regulation of endothelial cell function triggered by high glucose via the autophagy pathway (14).

To determine the roles of metformin in DN, a rat model of DM was established to examine whether and how metformin confers protection in DN induced by streptozotocin (STZ).

## MATERIALS AND METHODS

### Experimental animals

The experiments involving animals followed the institutional guidelines and were approved by the Animal Ethics Committee of Fujian Medical University.

Male Sprague Dawley rats (6 weeks old, 180 to 200 g), provided by Beijing SPF Biotechnology (China), were housed in individual cages under specific pathogen-free conditions, with a 12-hour photoperiod and freely available water in the Laboratory Animal Center of Fujian Medical University. After a 7-day adaptation, the animals were randomized to two groups. 1 – The normal chow group (NC, n = 10) received a standard chow diet (24% protein, 66% carbohydrates, and 10% fat) for 8 weeks, followed by oral administration of 0.9% saline for 8 weeks. 2 – The DM group (DM, n = 20) was fed HFD (20% protein, 20% carbohydrates, and 60% fat; H10060, Beijing HFK Bioscience, China) for 8 weeks, and then subjected to intraperitoneal STZ induction (30 mg/kg) using citrate buffer (pH 4.5). Tail vein blood collection was performed 72 hours after intraperitoneal treatment with STZ to measure blood glucose (BG) levels; BG > 16.7 mmol/L suggested successful DM modeling. The DM group was randomly subdivided into a DM control group (DMC, n = 10; HFD for 8 weeks and STZ treatment, followed by 0.9% saline administered orally for 8 weeks) and a metformin group (DMM, n = 10; HFD for 8 weeks and STZ treatment, followed by metformin 400 mg/kg [Sigma-Aldrich] administered orally in 0.9% saline daily for 8 weeks). The DMM and DMC groups received equal amounts of the vehicle 0.9% saline, with and without metformin, respectively. After the 16-week treatment period, euthanasia was performed by intraperitoneal treatment with sodium pentobarbital (40 mg/kg). Levels of BG were assessed every 4 weeks using a glucometer (Bayer, Leverkusen, Germany). Blood specimens were obtained by cardiac puncture. The experimental design and dosage regimens were based on previous reports of experimental DN (15,16).

### Tail-flick test

The rats’ tails were continuously irradiated with a light radiometer (Changchun New Industry Optoelectronic Technology, mdl-hd-635, China), and the time from the beginning of irradiation to the occurrence of a tail-flick reaction (tail-flick latency in seconds) was recorded. Each rat was tested thrice, and values were averaged. The upper limit of the incubation period was set at 30 seconds to avoid damaging the tails.

### Motor nerve conduction velocity

Sciatic nerves were stimulated with previously inserted electrodes using constant-current (10 to 20 mA) square-wave pulses (40 µs) for the generation of compound muscle action potential. A total of three latency-of-M wave pairs were obtained and averaged. The average latency difference (ALD) between the first-onset peak and the maximum negative peak was considered the conduction time between the two sites. Motor nerve conduction velocity was derived as the distance separating the stimulating electrodes divided by the ALD.

### Sensory nerve conduction velocity

Sciatic nerves were stimulated with previously inserted electrodes using constant-current (2 mA) square-wave pulses (40 µs) to evoke an H-reflex. Then, six latency pairs were obtained, to determine the minimal latency difference (MLD) between the two sites. Sensory nerve conduction velocity (SNCV) was derived as the distance separating the stimulating electrodes divided by the MLD. Stimulus digitization and capture utilized the RM6240 multi-channel signal collection system.

### Histology detection

Sciatic nerve segments (1 to 2 cm) underwent overnight fixation with 25 g/L glutaraldehyde at 4 °C. This was followed by a 1-hour post-fixation with 10 g/L osmium tetroxide at 4 °C, dehydration, resin embedding, and placement in an Araldite mixture. Blocks underwent polymerization at 60 °C for 48 hours. Semi-thin sections underwent staining with 5% uranyl acetate and lead citrate, followed by observation under an electron microscope (Hitachi, Japan) (17).

### Western blotting

Protein samples were obtained from sciatic nerves lysed with RIPA lysis buffer supplemented with protease inhibitors (Beyotime, China). Sodium dodecyl sulfate-polyacrylamide gel electrophoresis (SDS-PAGE) was performed for protein separation, followed by electro-transfer onto polyvinylidene fluoride membranes (Millipore, USA). Upon blocking with 5% skimmed milk, the membranes underwent overnight incubation at 4 °C with primary antibodies targeting p-AMPK (Thr-172), AMPK, LC-3, and β-actin (Cell Signaling Technology, USA), followed by chemiluminescent detection (18). ImageJ version 1.48 (NIH, USA) was utilized to quantify densitometric signals.

### Statistical analysis

Data are shown as mean ± standard error of the mean (SEM). Group pairs and multiple groups were compared with Student's *t* test and analysis of variance (ANOVA), respectively, using GraphPad Prism 8.0 (GraphPad, USA). P < 0.05 indicated statistical significance.

## RESULTS

### Metformin ameliorates blood glucose in the rat model of diabetes

**Figure 1:**
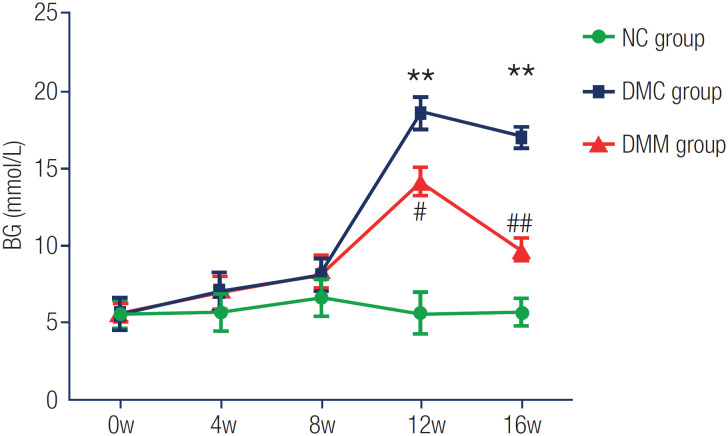
Blood glucose levels

No significant differences were found in baseline BG levels among the study rats. After 8 weeks of HFD, BG levels were slightly higher in rats of the DM group compared with those of the NC group, but with no significant difference (p > 0.05). However, STZ administration combined with HFD induced partial destruction of β cells in the islet, which decreased insulin secretion, inducing DM in the rat model. After metformin treatment, BG in the DMM group was controlled to a certain extent, with levels markedly reduced compared with the DMC group (p < 0.05), although still elevated compared with those of the NC group (Table 1 and Figure 1).

**Table 1:** Blood glucose levels

Week	NC group (mmol/L)	DMC group (mmol/L)	DMM group (mmol/L)
0w	5.61 ± 0.86	5.59 ± 0.92	5.76 ± 0.54
4w	5.68 ± 1.14	6.98 ± 1.09	6.89 ± 1.18
8w	6.03 ± 1.09	8.14 ± 1.01	8.26 ± 1.03
12w	5.55 ± 1.32	18.61 ± 0.87**	14.15 ± 0.86#
16w	5.69 ± 0.84	17.03 ± 0.63**	9.84 ± 0.57##

Data are shown as mean ± standard error of the mean (SEM). Abbreviations: DMC group, diabetes mellitus group; DMM group, metformin group; NC group, normal chow group; w, weeks.

** p < 0.01. *vs* NC;

# p < 0.05 vs. DMC;

## p < 0.01 vs. DMC.

Blood glucose curves across all groups. After 8 weeks of a high-fat diet, blood glucose levels were slightly higher in rats of the diabetes mellitus group compared with those in the normal chow (NC) group, but the difference was not statistically significant (p > 0.05). However, a single dose of streptozotocin injection combined with high-fat diet resulted in the occurrence of diabetes mellitus in the rat model. After metformin treatment, blood glucose in the metformin group (DMM) group was controlled to a certain extent. Blood glucose levels became significantly lower in the DMM group than in the DMC group (p < 0.05) but were still higher than those in the NC group. Data are expressed as mean ± standard error of the mean (SEM). Abbreviations: BG, blood glucose level; DMC group, diabetes mellitus group; DMM group, metformin group; NC group, normal chow group; w, weeks. *|*p < 0.01 vs. NC; #p < 0.05 vs. DMM; ##p < 0.01 vs. DMM.

### Metformin ameliorates peripheral nerve symptoms in diabetic rats

**Figure 2:**
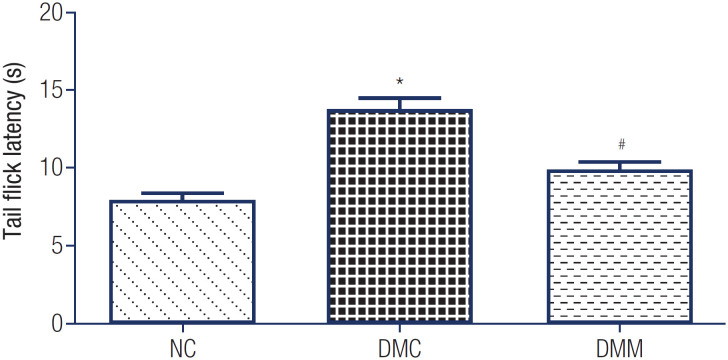
Tail-flick latency

The tail-flick test was applied to assess the level and change of the rats’ pain threshold and to evaluate neuropathic pain. As depicted in Figure 2, tail-flick latency was significantly longer in the DMC group compared with the NC group (13.55 ± 2.21 seconds *vs.* 7.76 ± 1.26 seconds, p < 0.01). However, after 10 weeks of metformin intervention, tail-flick latency was remarkably shorter in the DMM group compared with the DMC group (9.9 ± 0.85 seconds *vs.* 13.55 ± 2.21 seconds, p < 0.05).

Tail-flick test results, expressed as tail-flick latency across all groups. Tail-flick latency was significantly longer in the rats of the diabetes mellitus (DMC) group compared with those in the normal chow (NC) group (13.55 ± 2.21 seconds vs. 7.76 ± 1.26 seconds, respectively, p < 0.01). However, after 8 weeks of metformin treatment, tail-flick latency was significantly shorter in the metformin (DMM) group compared with the DMC group (9.9 ± 0.85 seconds vs. 13.55 ± 2.21 seconds, p < 0.05). Data are expressed as mean ± standard error of the mean (SEM). Abbreviations: NC group, normal chow group; DMC group, diabetes mellitus group; DMM group, metformin group; s, seconds. *p < 0.01 vs. DMC; #p < 0.05 vs. DMM.

Both motor nerve conduction velocity (MNCV) and SNCV were determined to examine the function of myelinated nerve fibers (Table 2). The conductance of motor and sensory nerves was significantly lower in the DMC group compared with the NC group. In contrast, MNCV and SNCV were higher in the DMM group compared with the DMC group (p < 0.05).

**Table 2:** Motor nerve conduction velocity and sensory nerve conduction velocity across groups

	NC group	DMC group	DMM group
MNCV (m/s)	59.81 ± 2.38	44.58 ± 4.19*	52.81 ± 3.56#
SNCV (m/s)	49.2 ± 2.14	36.51 ± 3.01**	42.08 ± 3.31#

Data are presented as mean ± standard error of the mean (SEM). Abbreviations: MNCV, motor nerve conduction velocity; SNCV, sensory nerve conduction velocity; NC group, normal chow group; DMC group, diabetes mellitus group; DMM group, metformin group; m/s, meters per second.

* p < 0.05 *vs.* NC;

** p < 0.01 *vs.* NC;

# p < 0.01 *vs.* DMM.

### Metformin ameliorates the quantity and morphology of myelinated fibers of the sciatic nerve in diabetic rats

**Figure 3:**
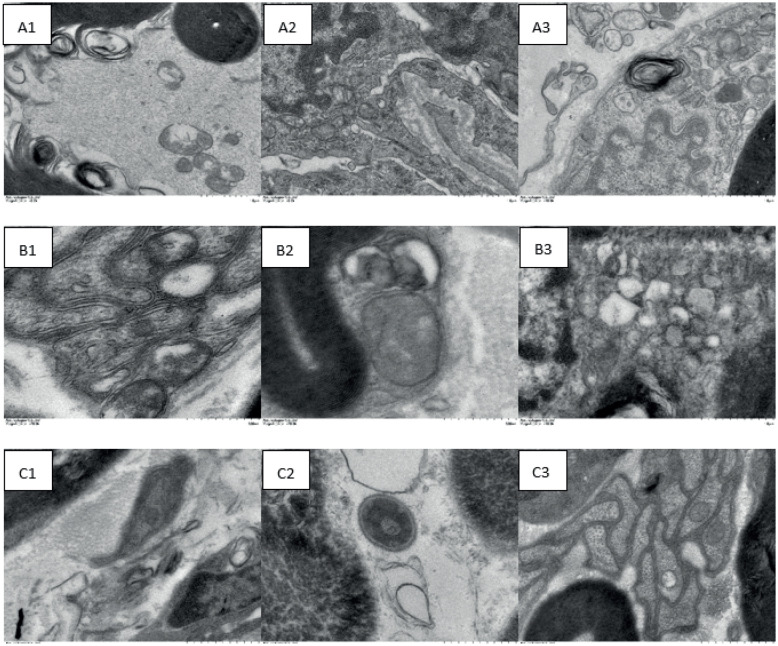
Effects of metformin on the number and morphology of myelinated fibers in the sciatic nerve

Nerve fibers were uniform and dense, with lamellae shaped into concentric circles in the NC group (Figure 3 A1-A3). The axons were swollen and had no shrinkage, with aligned neurofilaments and microtubules. Schwann cells and axonal mitochondria were not swollen. The ridge of the mitochondrial inner membrane was clearly visible, and myelin protrusion was scarce.

Electron micrographs of sciatic nerves from rats in the normal chow group (NC; A1, A2, and A3), diabetes mellitus control group (DMC; 81,82, and 83), and metformin group (DMM; C1, C2, and C3). After metformin treatment, myelinated nerve fibers had a relatively complete lamellar structure, with reduced density and uniformity. Occasionally, bubble-shaped defects, lamella separation, neurofilaments, and neurotubule disarrangement were observed, but the ridge of the mitochondrial inner membrane within the axon retained its original shape. Overall, demyelination and axonal lesions persisted, and the sciatic nerve pathology was substantially ameliorated in the DMM compared with the DMC group. Scale bar: 5 µm, 2 µm, or 0.5 µm.

Observations under the electron microscope revealed that sciatic nerves in the DMC group (Figure 3 B1-B3) had altered myelin configuration, myelin protrusion, lamella separation, neurofilaments, neurotubule accumulation and disarrangement, and various bubble-shaped defects in the myelinated axons. Schwann cells and axonal mitochondria were broadly swollen or lysed, and no ridge was observed in the mitochondrial inner membrane.

In the DMM group (Figure 3 C1-C3), myelinated nerve fibers had a relatively complete lamellar structure with reduced density and uniformity. Some bubble-shaped defects, lamella separation, neurofilaments, and neurotubule disarrangement were detected, but the ridge of the mitochondrial inner membrane regained its normal shape. In general, demyelination and axon damage persisted, although sciatic nerve pathology was considerably improved in the DMM group compared with the DMC group.

### Metformin inhibits autophagy in the sciatic nerve of diabetic rats

**Figure 4:**
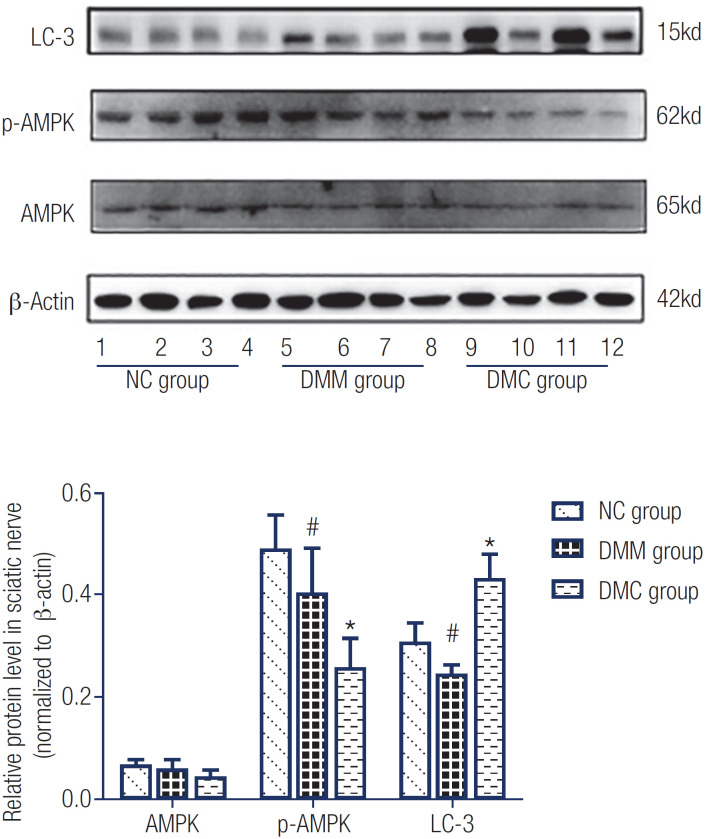
Autophagy, AMPK, and p-AMPK levels in the sciatic nerve

As depicted in Figure 4, relative AMPK expression levels were similar in the DMM (0.058 ± 0.02), DMC (0.045 ± 0.01), and NC (0.066 ± 0.01) groups. P-AMPK (Thr-172) expression was decreased in the DMC group compared with the NC group but restored after the metformin intervention. Expression of LC-3 (which reflects autophagy level) was markedly upregulated in the DMC group compared with the NC group. After the metformin intervention (DMM group), autophagy level decreased significantly relative to the DMC group.

Relative protein levels of AMPK, p-AMPK (Thr-172), and LC-3 in sciatic nerves. Compared with the normal chow (NC) group (0.066 ± 0.01), no significant changes were detected in relative AMPK expression in the metformin (DMM) group (0.058 ± 0.02) and control (DMC) group (0.045 ± 0.01). However, p-AMPK (Thr-172) levels in the DMC group were decreased, and p-AMPK activity was restored after metformin intervention. In addition, LC-3 expression was significantly higher in the DMC group compared with the NC group. After metformin treatment, autophagy level was significantly decreased in the DMM compared with the DMC group. Data are expressed as mean ± standard error of the mean (SEM). Each group included 10 rats. Abbreviations: DMC group, diabetes mellitus group; DMM group, metformin group; NC group, normal chow group. *p < 0.01 vs. NC; #p < 0.05 vs. DMC.

## DISCUSSION

The occurrence of DN is known as a prominent DM complication (19), and its prevalence may be decreased by continuing improvements in clinical examination and diagnostic methods (20). Hence, the present study aimed to explore the mechanisms underpinning metformin-related alleviation of DN. The study results demonstrated that metformin alleviated peripheral neuropathy in diabetic rats by suppressing autophagy via the AMPK pathway, providing evidence that metformin is a fundamental treatment target in DN.

Many pathways jointly contribute to the pathogenetic mechanism of DN. However, the precise regulatory factors involved remain undefined. Slowing the development of DN and preventing or even reversing its symptoms remain the top therapeutic strategies for this disorder.

Autophagy is a natural process in which damaged organelles and macromolecules are degraded. Due to its double-sided regulatory role, autophagy contributes to maintaining intracellular environmental homeostasis; a normal level of autophagy protects cells from environmental insults, but excessive and insufficient autophagy might cause disease (21). In recent years, a series of cell culture and animal studies have confirmed the close relationship between autophagy and DN (22,23), but the specific functional relationship between both remains unclear.

Yerra and cols. (24) showed that autophagy exerts neuroprotective effects by decreasing the buildup of damaged organelles and proteins in nerve cells. Compared with normal cultured cells, Neuro2a (N2a) cells cultured with high glucose had reduced autophagosome formation, with lower beclin-1 and LC-3-II protein levels. In other studies, Purkinje cells in the cerebellum of a 24-week STZ-induced DN rat model were degenerated, accompanied by progressive expansion of the axon ends, decreased autophagosome formation, reduced Lamp2 expression and LC-3 II/LC-3 II ratio, and increased aggregation of the p62 protein, which is specifically degraded by autophagy. Similarly, beclin-1 was remarkably downregulated in the sciatic nerve of STZ-induced DN rats (22).

However, different views exist among scholars. For example, using in vivo experiments, Towns and cols. (25) found that dorsal root ganglion neurons in STZ-induced DN rats had impaired mitochondrial function, increased apoptosis, enhanced autophagy, and increased number of autophagosomes co-located with the mitochondria in the neuronal cell body.

Metformin is the most common drug used in type 2 DM treatment. The main action of metformin is in the gut, and through a gut-liver crosstalk, there is an indirect effect on gluconeogenesis in liver (26). Meanwhile, growing evidence suggests metformin contributes to the regulation of aging and cancer development (27). Notably, AMPK was initially described as a suppressor of liver acetyl-CoA carboxylase and HMG-CoA reductase, which are major factors controlling the biosynthetic pathways of fatty acid and cholesterol (28). In addition, metformin exerts protective effects in diabetic retinopathy, whose mechanisms are associated with AMPK-dependent and AMPK-independent pathways (29). In the present study, we established that autophagy level in diabetic rats was enhanced after 8 weeks of metformin treatment. Although AMPK expression was not remarkably changed, autophagy level was decreased with increasing p-AMPK amounts, thus ameliorating DN symptoms. Meanwhile, metformin also improved the number of myelinated fibers in the sciatic nerve and reversed the morphological changes of the sciatic nerve in diabetic rats.

Metformin has implications for lactate homeostasis. The drug's influence on lactate metabolism is of particular interest, given the established role of lactate as a critical energy substrate in neuronal and glial cells, as highlighted in a review by Jha and Morrison (30). Metformin activates AMPK, potentially impacting lactate production and utilization by altering cellular energy dynamics (31). This interaction could be significant in conditions like DN, where lactate transporter MCT1 is crucial for nerve function (32). Further research is needed to clarify metformin's role in lactate metabolism within the nervous system, which could reveal new therapeutic targets for neurological disorders.

In summary, the present study unveiled a probable mechanism by which metformin may suppress autophagy in the sciatic nerve via an AMPK-dependent pathway that phosphorylates AMPK at Thr-172 to improve DN. Therefore, metformin can be clinically used beyond its basic anti-DM potential. Indeed, its beneficial effects on diabetic complications are still being discovered. Consequently, metformin is to be administered throughout the treatment process of DM in patients without contraindications, regardless of the presence or absence of diabetic complications. In an upcoming study, we will explore other potential mechanisms of metformin in DN control.

## Data Availability

the data that support the findings of this study are available on request from the corresponding author. The data are not publicly available due to privacy or ethical restrictions.
